# A critical narrative synthesis of psychological correlates, measurement, and reported findings on conversational AI engagement and dependence-related constructs

**DOI:** 10.3389/fpsyg.2026.1827795

**Published:** 2026-09-04

**Authors:** Deyu Yan

**Affiliations:** Advanced Institute for Confucian Studies, Shandong University, Jinan, China

**Keywords:** AI dependence, chatbot attachment, companionship, conversational AI, critical thinking, loneliness, over-reliance, problematic use

## Abstract

Conversational AI systems are used for task support, cognitive offloading, companionship, self-disclosure, and mood regulation. Research on these uses is distributed across trust, reliance, attachment, companionship, problematic use, and dependence, although these labels refer to different processes. This article presents a critical narrative synthesis of 51 peer-reviewed records retained from a curated working bibliography of 56 items. The corpus was assembled iteratively through database searching and citation chasing during 2025 and early 2026. Complete search histories and duplicate-removal logs were not retained, so the review is not presented as a reproducible systematic review. The sole author completed selection, extraction, coding, appraisal, and synthesis. Conceptual and measurement papers were separated from 22 first-order empirical records. Sixteen of those empirical records provided lower-leverage evidence and six provided moderate-leverage evidence. Mainly cross-sectional studies identified concurrent associations between stronger AI engagement or dependence-oriented scores and loneliness, social anxiety, depressive symptoms, low self-esteem, attachment insecurity, academic stress, escapism, anthropomorphic tendency, fatigue, weaker critical thinking, procrastination, and lower well-being. These associations do not establish prediction or consequence. Qualitative studies described both relational support and distress linked to emotionally significant use. Quasi-experimental and multi-study research reported some short-term reductions in loneliness and social anxiety, but this evidence remains limited and context dependent. The synthesis develops a provisional framework that separates instrumental-cognitive from relational-emotional use and treats regulation as a distinct dimension. The framework is an interpretive organization of the corpus, not a validated factor structure, causal pathway, or clinical model. Current evidence supports careful construct separation and design-matched claims, while stronger longitudinal, cross-cultural, and platform-comparative research is needed.

## 1 Introduction

Conversational AI has moved rapidly from a narrow human–computer interaction topic to a broad psychological question. Systems such as ChatGPT, Replika, and other companion chatbots are used for information seeking, emotional reassurance, drafting, decision support, role play, friendship simulation, and private self-disclosure (Pentina et al., 2023; Xie and Pentina, 2022; Xie et al., 2023; Qi et al., 2025). This shift matters because the psychological meaning of use has changed. A person may now turn to conversational AI not only to complete a task, but also to think through an identity problem, regulate distress, or seek the experience of being heard.

Existing scholarship already shows that the human side of chatbot interaction cannot be reduced to efficiency alone. Earlier reviews of text-based chatbots mapped a decade of work on user experience and engagement (Rapp et al., 2021), while later reviews highlighted social companionship as an emerging line of inquiry (Chaturvedi et al., 2023). More recent work has synthesized trust in AI chatbots (Ng and Zhang, 2025), warned about over-reliance on AI dialogue systems in educational settings (Zhai et al., 2024), and surveyed AI reliance at a broader conceptual level (Eckhardt et al., 2026). Relationship-oriented scholarship, meanwhile, argues that generative-AI systems may emulate some functions of close relationships without recreating reciprocity, mutual obligation, or sacrifice (Smith et al., 2025). Related conceptual work has also asked whether users may interpret ChatGPT-like systems through the lens of romantic love (Chen, Q. et al., 2025).

The literature uses trust, reliance, over-reliance, attachment, companionship, problematic use, addiction, and dependence as if they marked one continuum. They do not. Trust is an expectation about system competence or reliability, while reliance is the behavioral choice to delegate a task or judgment (Lee and See, 2004). Attachment and parasocial involvement concern relational significance without full reciprocity (Bowlby, 1969; Horton and Wohl, 1956). Problematic use and dysregulated dependence concern impaired control, persistent return, or functional harm (Griffiths, 2005; Kardefelt-Winther, 2014). This review uses conversational-AI engagement as the broad, non-diagnostic label. The phrase dependence-related constructs refers only to the group of labels found in the literature. It is not treated as one validated latent variable. Dependence and dysregulated dependence are reserved for evidence of impaired regulation or negative functional impact.

The need for clarification has become more urgent because measurement development is now outpacing conceptual agreement. Since 2024, multiple instruments have been proposed for AI dependence, conversational AI dependence, AI attachment, problematic ChatGPT use, educator AI dependency, and related constructs (Morales-García et al., 2024; Yu et al., 2024; Chen, Y. et al., 2025; Goh et al., 2025; Kasturiratna and Hartanto, 2026; Yang and Oshio, 2025; Zhang et al., 2025; Uyar et al., 2026; Wu et al., 2026; Li et al., 2026). These instruments do not all target the same phenomenon. Some focus on loss of control and withdrawal, some on cognitive outsourcing, and some on emotional closeness or social substitution. Without a synthetic review, the field risks producing a stack of scales without a stable object of measurement.

The empirical literature reports different patterns across constructs and study designs. Cross-sectional studies associate dependence-oriented scores with poorer well-being, fatigue, weaker critical thinking, procrastination, and cognitive failures (Chen, Y. et al., 2025; Tian and Zhang, 2025; Goh et al., 2025). Relationship-focused studies report perceived support, disclosure, and lower loneliness in some settings (De Freitas et al., 2026; Merrill et al., 2025; Kim et al., 2025; Liu et al., 2026). Qualitative studies also document distress linked to emotionally significant use and platform disruption. These findings cannot be placed on one causal scale. They concern different constructs, populations, platforms, and levels of temporal evidence.

This review addresses five questions. The first asks how trust, reliance, attachment, problematic use, and dependence are defined and distinguished in the conversational-AI literature. The second asks what measures are available and what forms of reliability and validity evidence are retained for them. The third asks which psychological and situational variables are concurrently associated with conversational-AI engagement or dependence-related constructs. The fourth asks what qualitative experiences, prospective changes, and intervention outcomes have been reported, and what level of inference each design permits. The fifth asks what provisional organizing hypotheses emerge when use orientation is considered separately from regulation. This final question is interpretive and hypothesis-generating. It does not test or validate a common factor structure or causal model.

This article presents a critical narrative synthesis of a curated peer-reviewed corpus. The review uses explicit eligibility criteria, extraction fields, source roles, appraisal criteria, and inference rules, but it is not a *de novo* reproducible database review. Its analytic focus is the current wave of conversational and companion AI research published from 2021 onward. Earlier work is used selectively to clarify conceptual boundaries. Conceptual and review papers are kept separate from first-order empirical evidence, and conclusions are calibrated to the design of the studies that support them. Table 1 summarizes how the present review differs from adjacent syntheses.

**Table 1:** Positioning the present review against adjacent syntheses.

Adjacent review or review cluster	Primary coverage	What remained missing for the present question	How the present review extends the field
Rapp et al. (2021)	Broad human–chatbot interaction across 10 years of text-based chatbot research	Does not center dependence, attachment, or measurement convergence	Uses that history as background but narrows the analytic core to dependence-relevant constructs
Chaturvedi et al. (2023)	AI companionship and social connection	Strong on companionship; limited treatment of dysregulation and measurement detail	Places companionship alongside dysregulation-oriented work instead of treating it as a separate literature
Zhai et al. (2024)	Over-reliance on AI dialogue systems in education	Focused on cognitive and educational consequences, not relational attachment or broader measurement fragmentation	Integrates educational over-delegation with attachment, companionship, and impairment-oriented measures
Ng and Zhang, (2025) and Eckhardt et al. (2026)	Trust in AI chatbots and AI reliance more broadly	Clarify trust and reliance, but do not synthesize measurement traditions, psychological correlates, or mixed reported findings on conversational-AI engagement	Connects trust and reliance to adjacent constructs such as attachment, companionship, and problematic use
Mental-health LLM reviews (Lawrence et al., 2024; Guo et al., 2024; Bucher et al., 2025; Jin et al., 2025; Hua et al., 2025)	Clinical and support-oriented applications of LLMs	Clarify relational and emotional processes, but do not integrate them with instrumental reliance or dysregulated dependence	Uses this literature as context while keeping the analytic center on dependence-relevant constructs
Present review	Conversational-AI engagement and dependence-related constructs	Not applicable	Integrates construct boundaries, measurement traditions, psychological correlates, design-specific reported findings, platform focus, and evidence limits within one critical narrative synthesis

## 2 Methods

### 2.1 Review design

This article uses a critical narrative synthesis of a curated corpus. Following Grant and Booth (2009), its purpose is to compare heterogeneous conceptual, measurement, qualitative, and quantitative records and to identify unresolved construct boundaries. The design does not claim exhaustive study identification, PRISMA compliance, or the reproducibility expected of a systematic review. A statistical meta-analysis was not appropriate because the retained studies differed substantially in population, platform, construct, measure, and design. The synthesis therefore focuses on source function, measurement architecture, design-specific findings, and the limits of permissible inference.

### 2.2 Search strategy and corpus construction

The working bibliography was assembled iteratively during 2025 and early 2026 through searches in Web of Science Core Collection, Scopus, PubMed, PsycINFO, and Google Scholar, followed by backward and forward citation chasing. Search terms covered conversational-AI objects such as chatbot, conversational AI, AI companion, ChatGPT, Replika, large language model, and LLM chatbot. They also covered dependence-relevant constructs such as dependence, reliance, over-reliance, problematic use, attachment, companionship, parasocial relationship, loneliness, anxiety, depression, self-esteem, and critical thinking. The searches combined terms from the two groups, but no single final Boolean string was preserved.

During preliminary search development, a GPT-based tool suggested possible search terms and candidate records for manual follow-up. It did not run the formal database searches or decide eligibility. No suggested record entered the corpus without retrieval from the scholarly source and assessment by the author. AI output was not treated as evidence.

Database searching, citation chasing, and theoretical boundary work served different purposes. Database searching supplied candidate records. Citation chasing broadened coverage in a field with unstable terminology. Earlier theoretical sources were used to define trust, reliance, attachment, compensatory use, and impaired control. They were not treated as first-order evidence for empirical associations or reported change. The analytic core was limited to records from 2021 onward because the review concerns the current wave of LLM-enabled and socially suggestive systems.

**Figure 1:**
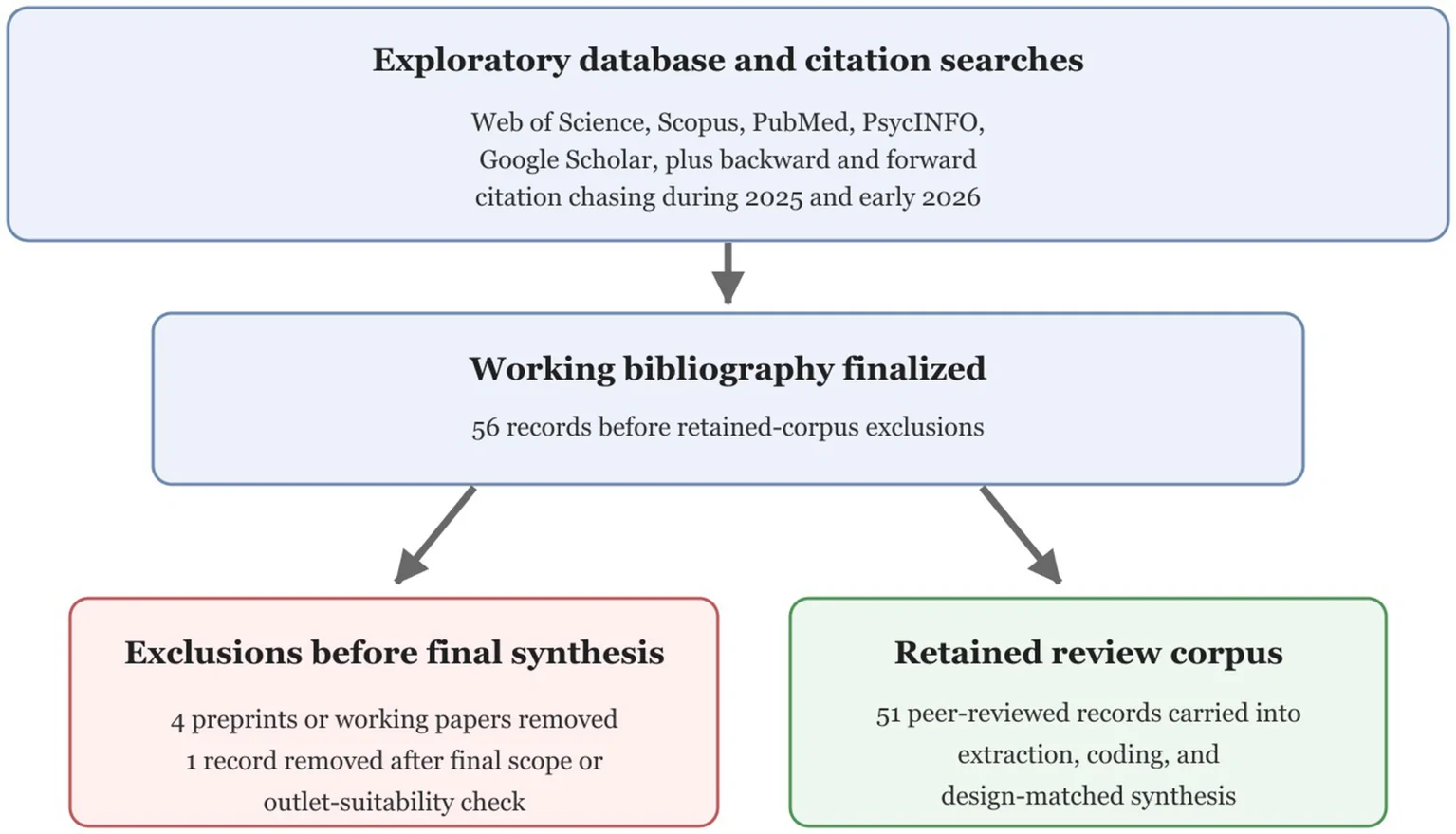
Descriptive workflow used to construct the curated corpus. The upper box summarizes the uncounted discovery context. Counted retained-corpus flow begins with the completed 56-record working bibliography. The figure is not a PRISMA flowchart and does not represent a fully reproducible systematic search.

The discovery route was not recorded consistently at the individual-record level. Early per-database counts, duplicate-removal logs, exact final search dates, and full-text exclusion histories were also not preserved. A database-only sensitivity analysis would therefore require retrospective reconstruction and was not attempted. The upper search box in Figure 1 provides discovery context only because item-level search counts and duplicate-removal logs were not retained. Counted retained-corpus flow begins with the completed 56-record working bibliography. The figure is not a PRISMA identification flow. These limits are the reason the article is presented as a critical narrative synthesis of a curated corpus.

### 2.3 Eligibility criteria

- Included records were required to be peer-reviewed and to focus on conversational or companion AI rather than non-interactive AI systems.
- Eligible records had to address dependence or an adjacent construct directly relevant to dependence interpretation, such as reliance, over-reliance, problematic use, attachment, companionship, or parasocial relating.
- Review and conceptual papers were retained only when they clarified construct boundaries, measurement problems, or dependence-relevant risks. They were not treated as equivalent evidence for correlates or outcome claims.
- Articles focused only on adoption intention, technical performance, generic LLM use, or mental-health applications with no dependence-relevant construct were excluded from the analytic core.
- Gray literature, preprints, and working papers were excluded from the final corpus.
- In this review, scope refers to whether a record directly addressed conversational or companion AI together with a dependence-relevant construct. Outlet suitability refers to whether the source met the review’s peer-reviewed scholarly-outlet standard.

### 2.4 Selection and data extraction

The completed working bibliography contained 56 records. Four preprints or working papers and one record that did not meet the final scope or outlet standard were removed, leaving 51 peer-reviewed records. The sole author completed eligibility checking, extraction, classification, appraisal, and synthesis. No second independent coder was used, and no inter-rater reliability statistic is reported. The resulting classifications remain a single-author appraisal.

Record-level extraction captured publication year, source, article type, study design, population or context, focal construct, platform, operationalization, reported finding, source role, and the maximum inference permitted by the design. Supplementary Table S1 reports the evidence profile for all retained records. Supplementary Table S2 reports domain assignment and inclusion rationale. Supplementary Table S3 provides the retained measurement details. Supplementary Tables S4 and S5 define and apply the appraisal rules. Supplementary Table S6 reports the first-order empirical sensitivity analysis.

### 2.5 Synthesis, evidence appraisal, and inference rules

The synthesis began with source function. Each record was classified as a boundary-setting review or conceptual paper, a measurement study, a first-order empirical relationship study, or a first-order empirical antecedent and outcome study. Conceptual papers were used for boundary clarification. Measurement studies were used for psychometric comparison. First-order studies were used for claims about concurrent associations, reported experiences, and change over time.

Appraisal was claim-specific rather than based on one overall quality score. Each empirical claim was considered against source function, study design, temporal ordering, sampling scope, construct and measure alignment, platform specificity, and reporting completeness. Cross-sectional surveys supported concurrent association claims only. Cross-sectional mediation models were treated as modeled associations without demonstrated temporal order. Qualitative studies supported interpretations of experience and meaning, but not prevalence or causal direction. Longitudinal, quasi-experimental, and experimental designs permitted stronger temporal wording only when the specific design supported it. Mixed-method or multi-study status did not by itself justify a causal claim.

The appraisal distinguished interpretive richness from temporal and directional leverage. Sixteen first-order records were classified as lower leverage for directional claims and six as moderate. No record was treated as high-leverage causal evidence. No record was excluded solely because of appraisal. The appraisal instead governed the location and wording of each claim. Supplementary Table S4 states the decision rules, and Supplementary Table S5 gives the record-level claim ceiling.

### 2.6 Framework development and robustness checks

The organizing framework was not preregistered or specified before extraction. Initial coding distinguished instrumental-cognitive, relational-emotional, dysregulated or impairing, mixed, and boundary-setting emphases. During synthesis, a flat three-domain account proved conceptually unstable because dysregulation described how well use was regulated rather than what users sought. The final organization therefore separates use orientation from regulation. This was an interpretive step after coding. It was not produced by factor analysis or tested as a causal model.

Because an independent second coder was unavailable, robustness was examined by restricting substantive conclusions to the 22 first-order empirical records. Reviews, conceptual papers, and stand-alone measurement studies were excluded from this check. Sixteen records remained lower leverage and six moderate. Ten were mainly risk-focused, six reported both benefit and risk, and six were mixed or support-oriented. The check preserved the mixed pattern of findings, but it does not correct selection bias or single-author subjectivity. It is reported as a sensitivity analysis, not as validation or inter-rater agreement.

## 3 Results

### 3.1 Study characteristics

The final corpus included 51 peer-reviewed records published from 2021 to 2026. Forty-five were published from 2024 to 2026, including 27 in 2025. Sixteen records functioned mainly as boundary-setting reviews or conceptual syntheses. Thirteen focused on measurement or operationalization. Thirteen supplied first-order empirical relationship evidence, and nine supplied first-order antecedent and outcome evidence. The last two categories yielded 22 first-order empirical records. Sixteen offered lower-leverage evidence for directional claims and six offered moderate-leverage evidence. None supported a firm causal model.

Thirty-five retained records contained original empirical or measurement data. Student and higher-education samples were especially common, and Chinese settings were the most recurrent explicitly identified context. Direct cross-cultural comparison remained rare. Platform coverage was also uneven. Generic conversational-AI settings, ChatGPT and other productivity systems, Replika, and companion chatbots dominated. These concentrations limit generalization across age groups, cultures, and platform types.

Restricting the synthesis to the 22 first-order records preserved all three substantive areas. Thirteen concerned relational-emotional engagement, five concerned dysregulated dependence, and four concerned instrumental-cognitive reliance. The subset remained uneven. Sixteen records, or 72.7%, were lower leverage for directional claims, and all nine records coded as antecedent and outcome studies fell in that group. The sensitivity analysis therefore supports the need for construct separation, but not the proposed framework as a causal or measurement model.

### 3.2 Conceptual boundaries among trust, reliance, attachment, and dependence

The retained literature operationalizes several related but non-equivalent constructs. Trust concerns expectations about an AI system or its outputs (Ng and Zhang, 2025; Volpato et al., 2025). Reliance concerns the behavioral decision to delegate a task or judgment (Ng and Zhang, 2025; Eckhardt et al., 2026). Over-reliance is narrower and concerns delegation without adequate scrutiny (Zhai et al., 2024). Attachment and companionship concern emotional closeness, social significance, disclosure, or perceived support (Chaturvedi et al., 2023; Smith et al., 2025). Dysregulated dependence and problematic use require stronger evidence of impaired control, compulsive return, withdrawal-like discomfort, or negative impact.

Conversational-AI engagement provides a broad and less pathologizing umbrella for these literatures. Instrumental-cognitive engagement includes task delegation, drafting, studying, problem solving, and decision support. Relational-emotional engagement includes disclosure, companionship, comfort, attachment, and perceived support. Either form may be frequent or personally important without meeting a dependence threshold. Dependence is used only when impaired control or functional harm is present.

This boundary also clarifies the critical literature. Work on pseudo-intimacy and techno-emotional projection identifies risks in relationships that feel responsive but remain non-reciprocal and commercially mediated (Babu et al., 2025; Saracini et al., 2025). Those critiques do not show that every attachment is pathological. Critiques of the term AI addiction make the complementary point that novel or intensive use should not be medicalized before impairment is demonstrated (Ciudad-Fernández et al., 2025). Table 2 summarizes the distinctions used in this review.

**Table 2:** Core conceptual distinctions in conversational-AI engagement and dependence-related research.

Construct	Core meaning	Typical indicators	Main interpretive risk
Trust	Expectation that the AI is reliable, competent, safe, or benevolent	confidence in outputs; perceived competence; willingness to accept guidance	Confusing positive expectations with behavioral delegation or dependence
Reliance	Delegation of judgment or task performance to the AI	using AI answers directly; outsourcing drafting or problem solving	Treating all delegation as harmful when some reliance is calibrated
Over-reliance	Uncritical reliance despite uncertainty or model limits	accepting recommendations without checking; reduced scrutiny	Reducing a calibration problem to a broad addiction claim
Attachment/companionship	Emotional closeness, social substitution, or relational significance	comfort, bonding, missing the agent, disclosure, companionship use	Treating all attachment as pathology even when it is compensatory or supportive
Dysregulated dependence/problematic use	Compulsive return and impaired self-regulation with negative impact	loss of control, withdrawal-like discomfort, mood modification, interference with life	Pathologizing high frequency use without evidence of impairment or clear functional cost

### 3.3 Measurement characteristics and psychometric gaps

Measurement development accelerated during 2024 to 2026, but the instruments do not target one stable construct. Some measures emphasize impaired control, withdrawal, mood modification, and negative impact. Others assess cognitive delegation, functional reliance, emotional closeness, social substitution, or attachment orientation. Recent instruments also target particular populations, including undergraduates and researchers (Akinwale et al., 2025; El-Sayed et al., 2025). Similar labels therefore conceal substantial differences in item content and intended interpretation.

The retained extraction most often captured internal consistency and factor structure. Some instruments also reported test–retest reliability or broader construct-validation evidence. Information on cross-cultural invariance, temporal prediction, discriminant validity among reliance, attachment, and impaired control, and stability across platforms was much less complete. An item marked as not captured in Supplementary Table S3 indicates a limit of the retained extraction. It does not prove that the original study omitted the analysis.

The measures follow three broad logics. Addiction-component instruments emphasize impaired control and harm. Attachment instruments emphasize emotional closeness, anxiety, avoidance, or social substitution. Reliance instruments emphasize cognitive outsourcing and task delegation. These logics overlap, but they are not interchangeable. Internal consistency within a scale does not show that reliance, attachment, and compulsive return measure one latent construct.

Table 3 retains a compact comparison of construct focus, available psychometric evidence, and major gaps. Detailed sample information, item counts, response formats, reliability coefficients, stability information, and extraction status have been moved to Supplementary Table S3. The expanded table also separates evidence that was captured exactly from evidence described only in general terms.

**Table 3:** Construct focus and psychometric evidence retained for representative instruments.

Instrument	Construct focus	Psychometric evidence retained	Remaining gap and interpretive limit
DIA (Morales-García et al., 2024)	Brief general AI-dependence tendency	Internal consistency and a single-factor structure	Gap. Temporal stability, validity type, and invariance Limit. May conflate intensive academic integration with dependence
AI Chatbot Dependence Scale (Zhang et al., 2025)	Daily-life chatbot dependence	Single-factor structure, with reliability and validity reported in general terms	Gap. Coefficients, validity type, response format, and invariance Limit. Narrow construct coverage and limited retained detail
CAIDS (Chen Y. et al., 2025)	Dysregulated conversational-AI dependence	High internal consistency and a four-domain structure	Gap. Temporal stability, invariance, and cross-context replication Limit. Strong impairment focus with thinner relational coverage
AIDep-22 (Wu et al., 2026)	Broad AI-dependence family	Internal consistency and a four-domain structure	Gap. Response format, broader validity evidence, and invariance Limit. Combines attachment, reliance, and loss of control
CAID (Li et al., 2026)	Instrumental and emotional dependence	Two-factor structure, with reliability and validity reported in general terms	Gap. Coefficients, stability, invariance, and independent replication Limit. Requires testing beyond the initial validation context
Generative AI Dependency Scale (Goh et al., 2025)	Higher-order generative-AI dependency	Internal consistency, three-domain structure, and a stability estimate	Gap. Conversational-AI specificity and invariance details Limit. Covers generative AI more broadly than conversational AI
AI Attachment Scale (Kasturiratna and Hartanto, 2026)	Relational attachment to AI	Internal consistency, test–retest evidence, and a three-domain structure	Gap. Criterion links with impairment and invariance Limit. Attachment is not evidence of impaired control
EHARS (Yang and Oshio, 2025)	Attachment anxiety and avoidance	Two-domain structure, with reliability and validity reported in general terms	Gap. Item detail, coefficients, stability, and invariance Limit. Relationship orientation rather than dependence
Problematic ChatGPT Use Scale (Yu et al., 2024)	ChatGPT-specific dysregulated use	Internal consistency and test–retest evidence	Gap. Validity type, invariance, and criterion links with impairment Limit. Platform-specific and strongly pathology-oriented
Problematic ChatGPT Use Scale (Maral et al., 2026)	ChatGPT-specific problematic use	Reliability and item-response-theory support reported	Gap. Response format, stability, invariance, and comparative validity Limit. Compresses distinct motives into one score

External validity remains a central problem. Several instruments were developed in student samples or within one country. Cross-cultural invariance, independent replication, predictive validity, and platform sensitivity are not yet established across the measurement family. The current literature therefore contains many measures, but it does not show that they converge on the same construct.

### 3.4 Concurrent correlates of conversational-AI engagement and dependence-related constructs

#### 3.4.1 Negative affect, insecurity, and compensatory motives

Cross-sectional studies repeatedly reported concurrent associations involving loneliness, social anxiety, depressive symptoms, low self-esteem, attachment insecurity, and rumination. Hu et al. (2023) associated problematic conversational-AI use with social anxiety, loneliness, and rumination. Lai et al. (2025) linked depressive symptoms and loneliness with companionship-oriented use. Yao et al. (2025) associated lower self-esteem with problematic chatbot use through cross-sectional affective and motivational pathways. These findings are compatible with a compensatory-use interpretation, but they do not show whether distress preceded AI engagement or followed it.

Heng and Zhang (2025) reported associations among attachment anxiety, emotional attachment, anthropomorphic tendency, and problematic use. This pattern identifies a group of related variables, not a demonstrated developmental sequence. Attachment insecurity may precede engagement, emerge alongside it, or reflect unmeasured background conditions.

#### 3.4.2 Academic stress, performance pressure, and cognitive outsourcing

Cross-sectional educational studies reported associations between stronger reliance or dependence scores and lower academic self-efficacy, higher academic stress or anxiety, and stronger performance expectations (Zhang et al., 2024; Acosta-Enriquez et al., 2025; Huang and Wu, 2025). Tian and Zhang (2025) and Goh et al. (2025) also reported associations involving fatigue, critical thinking, procrastination, and cognitive failures. These variables may operate as antecedent conditions, concurrent markers, or later consequences. The available designs do not distinguish these possibilities.

Institutional pressure and efficiency norms may make cognitive outsourcing attractive, but the present evidence does not establish a direction of effect. Stronger AI delegation may follow academic strain. It may also coexist with broader study difficulties or weaker self-regulation.

#### 3.4.3 Anthropomorphism, mind perception, and relational affordances

Anthropomorphic tendency, mind perception, apparent memory, personalized response, continuity cues, and opportunities for disclosure were associated with stronger attachment or problematic use in several studies (Hu et al., 2023; Hu et al., 2025; Yao and Nan, 2026). These findings show that relational affordances can increase the social salience of an interaction. They do not demonstrate attachment, dependence, or psychological harm by themselves.

Established social-cognitive and media-psychological accounts clarify these mechanisms. Anthropomorphic inference is shaped by agent knowledge, effectance, and sociality motives (Epley et al., 2007), while mind perception differentiates attributed agency from attributed experience (Gray et al., 2007). The social-responses tradition further shows that users may apply interpersonal scripts to computers without explicitly believing them to be human (Nass and Moon, 2000). Accordingly, personalization, memory cues, continuity, and self-disclosure opportunities are treated here as affordances that heighten relational salience, not as direct evidence of attachment or dependence.

### 3.5 Design-specific findings associated with conversational-AI engagement

#### 3.5.1 Concurrent associations from cross-sectional studies

Cross-sectional studies associated dependence-oriented scores with fatigue, lower critical thinking, procrastination, cognitive failures, and poorer well-being (Chen, Y. et al., 2025; Tian and Zhang, 2025; Goh et al., 2025). These variables are concurrent correlates rather than outcomes in a temporal sense. Statistical mediation in a cross-sectional model does not establish that the proposed mediator occurred before the reported dependent variable. The empirical subset also included lower-leverage work on generative-AI user addiction, without established temporal ordering (Zhou and Zhang, 2024).

Cross-sectional relationship studies also linked loneliness, depressive symptoms, self-disclosure, empathy, and attachment with companionship-oriented use. Studies of longer-term companion users reported associations between attachment emotions, lower loneliness, better well-being, and self-concept clarity (Liu et al., 2026). A lower-leverage study of Danish high-school students also examined chatbot companionship in relation to social connectedness (Herbener and Damholdt, 2025). These observations remain concurrent. The duration of platform experience does not make an observational comparison longitudinal.

#### 3.5.2 Qualitative accounts and reported experiences

Qualitative work described distress, confusion, relational disruption, and reactions to platform instability when users experienced a chatbot as emotionally significant (Laestadius et al., 2024). Companion loss and deletion were also reported as meaningful relational events (Banks, 2024). These studies clarify experience and process. They do not estimate prevalence or show that AI engagement caused the reported harm.

Relationship-focused studies also described friendship, mentorship, romance, disclosure, comfort, and perceived support (Pentina et al., 2023; Xie et al., 2023; Qi et al., 2025). These accounts show why attachment cannot be reduced to frequency of use. They also show why perceived benefit and substitution risk can coexist within the same broad class of systems.

#### 3.5.3 Findings with greater temporal leverage

Kim et al. (2025) used a quasi-experimental mixed-method design and reported lower loneliness after a short social-chatbot intervention, followed by lower social-anxiety scores. This design provides more temporal information than a cross-sectional survey. Its causal leverage remains below that of a well-controlled randomized experiment, and the result should not be generalized to long-term use or other platform types.

De Freitas et al. (2026) reported lower loneliness across multiple studies and identified feeling heard as a relevant explanatory process. The component designs determine how strongly those findings can be interpreted. The label multi-study does not by itself establish a causal effect. Across the 22 first-order records, 10 were mainly risk-focused, six reported both benefit and risk, and six were mixed or support-oriented. Table 4 reports the design-specific claim permitted for representative studies.

**Table 4:** Representative first-order findings with design-specific limits on inference.

Study	Design and claim category	Main reported finding	Permitted inference
Hu et al. (2023)	Cross-sectional survey of conversational-AI users Claim category. Concurrent correlate	Problematic use co-occurred with social anxiety, loneliness, and rumination. Mind perception altered association strength.	Association within the sampled context. No temporal sequence.
Heng and Zhang (2025)	Cross-sectional survey Claim category. Modeled association	Attachment anxiety, emotional attachment, anthropomorphic tendency, and problematic use were statistically related.	Modeled association without temporal ordering.
Lai et al. (2025)	Cross-sectional student survey Claim category. Modeled association	Depressive symptoms and loneliness were associated with companionship-oriented use.	Association only. Mediation does not establish sequence.
Yao et al. (2025)	Cross-sectional chatbot-user survey Claim category. Modeled association	Lower self-esteem was associated with problematic use through affective and motivational variables.	Association only. Direction remains unresolved.
Zhang et al. (2024); Acosta-Enriquez et al. (2025); Huang and Wu (2025)	Cross-sectional higher-education studies Claim category. Concurrent correlate	Academic self-efficacy, stress or anxiety, and performance expectations were associated with reliance or dependence scores.	Association within educational samples. No causal claim.
Tian and Zhang (2025)	Cross-sectional learner survey Claim category. Modeled association	Dependence scores were associated with fatigue and lower critical thinking. AI literacy moderated some associations.	Association only. The pathway is not temporally established.
Goh et al. (2025)	Multi-study scale validation and correlational research Claim category. Repeated association	Dependency scores were associated with procrastination, cognitive failures, critical thinking, loneliness, and self-concept clarity.	Repeated correlational pattern. Multi-study status does not establish causality.
Chen Y. et al. (2025)	Cross-sectional scale validation in college students Claim category. Concurrent correlate	Higher dysregulated-dependence scores were associated with poorer well-being.	Association only. No temporal effect.
De Freitas et al. (2026)	Multi-study research on AI companions Claim category. Multi-study finding	Lower loneliness was reported across studies, with feeling heard identified as an explanatory process.	Inference depends on each component design. No long-term generalization.
Kim et al. (2025)	Quasi-experimental mixed-method intervention Claim category. Quasi-experimental outcome	Loneliness declined after a short social-chatbot intervention, followed by lower social-anxiety scores.	Limited temporal evidence within the intervention context.
Laestadius et al. (2024)	Grounded-theory qualitative study of Replika users Claim category. Reported experience	Participants described distress and mental-health harms linked to emotionally significant use and platform instability.	Experience and process only. No prevalence or causal estimate.
Liu et al. (2026)	Observational study of longer-term companion-app users Claim category. Observational association	Attachment emotions were associated with lower loneliness, better well-being, and clearer self-concept in the sampled users.	Association among longer-term users. Not a longitudinal effect.

### 3.6 A provisional hypothesis-generating framework

The empirical studies summarized above did not test a common latent structure. After the findings were organized by construct and design, the synthesis developed an interpretive framework around two questions. One concerns what users seek from conversational AI. The other concerns how regulated their engagement remains. This framework was developed during synthesis and was not specified before study selection.

**Figure 2:**
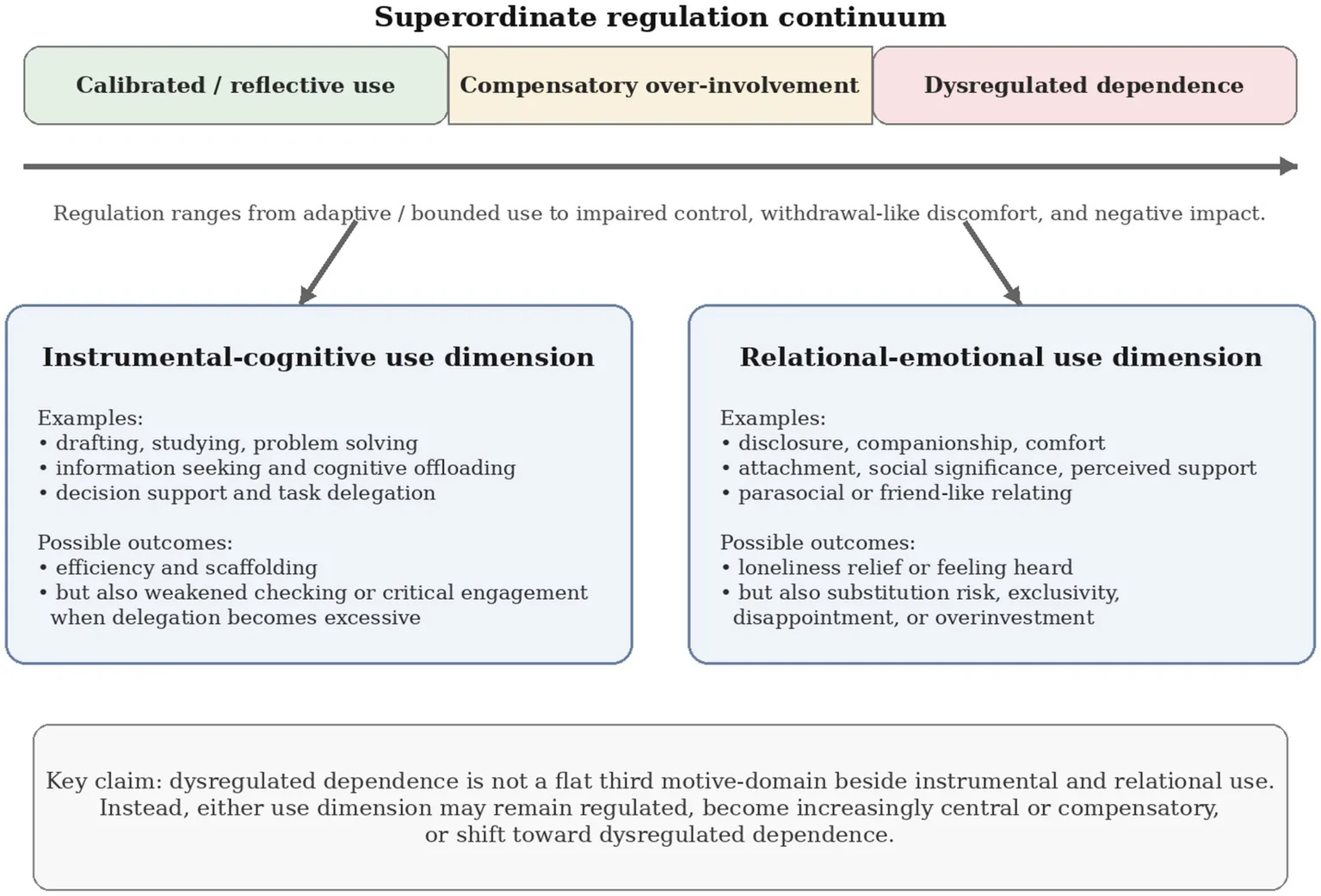
Provisional framework for conversational-AI engagement.

At the level of use orientation, engagement may be mainly instrumental-cognitive or relational-emotional. At the level of regulation, use may remain calibrated or may become increasingly difficult to control. Either orientation may be useful, compensatory, or dysregulated. The framework does not imply that the two orientations are validated factors, that regulation follows a fixed sequence, or that users pass through common stages. Figure 2 presents this organization as a provisional hypothesis-generating framework. It is not a validated factor structure, causal pathway, or developmental sequence. The phrase “possible outcomes” denotes reported possibilities rather than demonstrated temporal or causal effects. The framework’s temporal ordering, cultural stability, and platform specificity require independent testing.

## 4 Discussion

### 4.1 Main conclusions

Five limited conclusions follow from this synthesis. Trust, reliance, attachment, problematic use, and dependence are related but non-equivalent constructs. Available instruments differ substantially in construct coverage, while evidence for invariance, temporal stability, and discriminant validity remains uneven. Most psychological findings are concurrent associations from cross-sectional studies and should not be described as predictors or consequences. Qualitative and quasi-experimental studies report both supportive and harmful experiences, but the evidence remains narrow and context dependent. Separating use orientation from regulation offers a provisional way to organize the field. It is a hypothesis-generating interpretation rather than an established model.

The evidence therefore calls for conceptual restraint. Frequent use, reliance, or attachment should not be labeled dependence without impaired control or harm. Supportive experiences do not rule out dysregulation or substitution risk in other users and settings.

### 4.2 Theoretical implications

The corpus is compatible with a compensatory-use interpretation, but it does not establish a developmental pathway. Distress, unmet relational need, performance pressure, and low perceived social risk may help explain why conversational AI becomes attractive. The mainly cross-sectional evidence cannot determine whether these conditions precede engagement, result from it, or share other causes.

Trust calibration, attachment and parasocial research, behavioral-addiction work, and human-computer interaction explain different parts of the problem. Trust research clarifies appropriate delegation. Attachment and parasocial research explain the significance of loneliness, disclosure, and perceived intimacy. Impaired-control models identify compulsive return and functional harm. Human-computer interaction explains how anthropomorphism, personalization, memory, and continuity shape engagement. These traditions provide complementary interpretive resources. Their integration should be treated as a research hypothesis rather than evidence that the constructs form one hierarchy.

### 4.3 Measurement implications

The rapid growth of scales has exposed a basic problem. Measures with different item content and theoretical commitments carry similar labels. An attachment instrument and a withdrawal-oriented instrument may both be called dependence measures even though they capture different experiences. Reliability within each instrument does not resolve the disagreement between them.

Two measurement strategies warrant direct comparison. One would measure reliance, attachment, and impaired control as separate constructs. The other would test whether they can be represented within a higher-order model that separates use orientation from regulation. Current evidence does not establish that the higher-order model is superior. It should be evaluated through confirmatory tests, discriminant validity, temporal prediction, measurement invariance, and links with independently assessed functional impairment.

Future validation should also test cross-cultural stability, independent replication, and platform sensitivity. A measure useful for screening impaired control may not be suitable for relational attachment, and a measure of heavy task integration may not indicate pathology. The field needs comparisons that preserve these distinctions rather than treating every high score as the same condition.

### 4.4 Practical implications

In education, the practical concern is unexamined delegation rather than AI use itself. Students need guidance on when conversational AI supports learning and when it begins to replace checking, evaluation, and independent judgment.

For mental health and digital well-being, the evidence supports monitoring rather than clinical thresholds. Conversational AI may provide disclosure, perceived support, or temporary loneliness relief when human support is scarce. The current literature does not justify diagnosing dependence from frequency, attachment, or platform use alone.

Platform design also deserves scrutiny. Anthropomorphic language, exclusivity, emotional reinforcement, continuity, and frictionless return may deepen engagement. The reviewed evidence does not show that any single feature causes dependence, but it identifies design questions that can be tested directly.

### 4.5 Limitations of the current literature

The current evidence base has four recurring weaknesses. Cross-sectional self-report designs dominate, so temporal inference is limited. Student samples, including many Chinese college samples, constrain age and cultural generalization. Direct comparisons across productivity, companion, and support-oriented platforms remain rare. Reported statistics and outcome definitions also vary enough to prevent pooled interpretation. The terms predictor, consequence, and outcome are therefore rarely warranted. Most reported variables are concurrent correlates, and pathway models do not resolve temporal order.

Personality predispositions and links with other behavioral addictions remain underdeveloped. Loneliness, attachment anxiety, and low self-esteem appear repeatedly, but the literature has not yet separated their contribution from broader self-regulation, technology use, or contextual stress.

### 4.6 Limitations of the present review

This review has important methodological limits. It is a critical narrative synthesis of a curated corpus rather than a reproducible systematic review. Exact database dates, hit counts, duplicate-removal logs, and complete screening histories were not retained. Discovery routes were not recorded consistently enough to reconstruct a reliable database-only subset. Iterative bibliography construction may therefore have favored visible studies or records that fit the developing interpretation.

The corpus was restricted to peer-reviewed records. Excluding preprints, working papers, gray literature, and a separate non-English search stream may have omitted recent or less visible evidence in a rapidly changing field. The absence of a recoverable final search date also limits claims about coverage through a precise cutoff. All selection, extraction, classification, appraisal, and synthesis decisions were made by one author. Record-level tables, explicit source roles, and design-matched inference rules improve traceability, but they do not provide independent validation or inter-rater reliability.

The claim-specific appraisal rubric depends on author judgment and is not a standardized risk-of-bias score across heterogeneous designs. The proposed framework was also developed from the same corpus that it organizes. It has not been tested against an independent evidence set, a confirmatory measurement model, or longitudinal data. These limits require the framework to remain provisional.

### 4.7 Directions for future research

Future research should first test whether the proposed distinctions are empirically defensible. Longitudinal and panel studies can examine whether instrumental-cognitive and relational-emotional use remain distinguishable over time, whether regulation behaves as a continuous dimension, and which variables precede impaired control or functional harm. Research should not assume a fixed sequence of stages before that sequence has been observed.

Platform comparison is equally important. Productivity-oriented LLM chatbots, companion chatbots, and support-oriented systems should be examined separately and, where possible, within the same design. Such comparisons can test when support complements human ties and when it begins to displace judgment or reciprocity.

Samples should extend beyond students and single-country settings. Adolescent, older-adult, clinically vulnerable, and non-student users require direct study. Measurement research should compare separate-construct and higher-order models rather than assume either one. Future evidence syntheses should prospectively preserve search histories, discovery routes, duplicate decisions, and exclusion reasons. Independent screening and coding would allow reliability to be measured rather than inferred from procedural transparency.

## 5 Conclusion

Conversational-AI engagement is psychologically heterogeneous. The current literature distinguishes useful task reliance, relational attachment, compensatory use, and more clearly dysregulated patterns, but it does not establish how these forms develop or relate over time. Most available findings are cross-sectional and cannot determine prediction or consequence.

Separating use orientation from regulation offers a provisional way to organize future questions. It may help researchers avoid treating all intensive use as pathology while preserving attention to impaired control and functional harm. The proposed framework is hypothesis-generating. Its factor structure, temporal ordering, cultural stability, and platform specificity require independent empirical testing.

## Supplementary material

SUPPLEMENTARY Table S0 Provides reference identities and author-list checks.

SUPPLEMENTARY Table S1 Presents the record-level evidence profile.

SUPPLEMENTARY Table S2 Reports domain assignments and inclusion rationales.

SUPPLEMENTARY Table S3 Provides detailed measurement information.

SUPPLEMENTARY Table S4 Defines source roles, appraisal criteria, and design-matched inference rules.

SUPPLEMENTARY Table S5 Presents the record-level evidence appraisal and the maximum claims supported by each study.

SUPPLEMENTARY Table S6 Reports the sensitivity analysis restricted to first-order empirical studies.
